# Balancing the Efficiency and Sensitivity of Defect Inspection of Non-Patterned Wafers with TDI-Based Dark-Field Scattering Microscopy

**DOI:** 10.3390/s24051622

**Published:** 2024-03-01

**Authors:** Fei Yu, Min Xu, Junhua Wang, Xiangchao Zhang, Xinlan Tang

**Affiliations:** 1 Shanghai Engineering Research Center of Ultra-Precision Optical Manufacturing, School of Information Science and Technology, Fudan University, Shanghai 200438, China; 2 Shanghai Frontiers Science Research Base of Intelligent Optoelectronics and Perception, Institute of Optoelectronic, Fudan University, Shanghai 200438, China

**Keywords:** optical inspection, non-patterned wafer inspection, time delay integration, dark-field microscopy

## Abstract

In semiconductor manufacturing, defect inspection in non-patterned wafer production lines is essential to ensure high-quality integrated circuits. However, in actual production lines, achieving both high efficiency and high sensitivity at the same time is a significant challenge due to their mutual constraints. To achieve a reasonable trade-off between detection efficiency and sensitivity, this paper integrates the time delay integration (TDI) technology into dark-field microscopy. The TDI image sensor is utilized instead of a photomultiplier tube to realize multi-point simultaneous scanning. Experiments illustrate that the increase in the number of TDI stages and reduction in the column fixed pattern noise effectively improve the signal-to-noise ratio of particle defects without sacrificing the detecting efficiency.

## 1. Introduction

Defect inspection is essential in semiconductor manufacturing, especially in the production of non-patterned wafers, to ensure high-quality integrated circuits. The demand for smaller, faster, and more powerful electronic devices is increasing. As a result, the requirements for wafer inspection are becoming more stringent in terms of efficiency and sensitivity. However, achieving both high efficiency and high sensitivity simultaneously is a significant challenge due to mutual constraints [1,2]. Several techniques for wafer inspection, such as scanning electron microscopy (SEM) [3,4], atomic force microscopy [5,6], and confocal microscopy [7], exhibit excellent sensitivity but have relatively low throughput. In-line inspection systems, typically comprising optical wafer inspection instruments and SEM-based review instruments, are deployed at wafer production sites for process monitoring [8,9]. Optical wafer inspection instruments, such as bright-field microscopy and dark-field microscopy, enable rapid defect inspection through multi-channel acquisition and automated synchronized control. The subsequent defect review employs SEM to analyze the defects based on the defect coordinates coarsely located by optical inspection instruments. This process provides more comprehensive information about defects.

**Figure 1:**
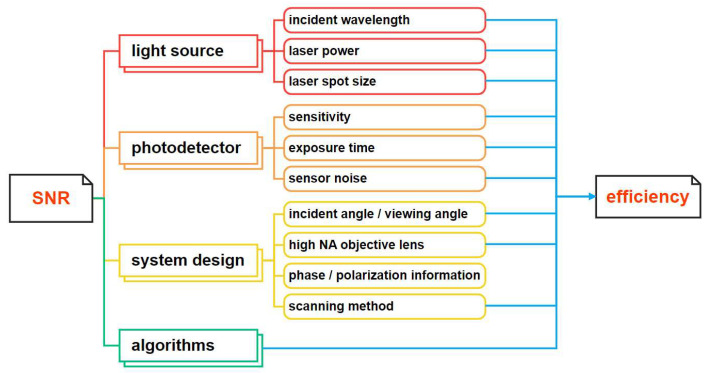
Factors affecting the SNR and detection efficiency.

Dark-field microscopy is the primary technique in non-patterned wafer inspection. Detection sensitivity highly depends on the signal-to-noise ratio (SNR) and image contrast of defects [10]. Figure 1 summarizes relevant factors affecting the SNR and efficiency. To enhance sensitivity, one can improve the light source with higher power and shorter wavelength. In practice, to ensure detection sensitivity, deep ultraviolet light sources with a wavelength of 248 nm or 193 nm and a power of several watts or even tens of watts are utilized. This also reduces exposure time thereby improving efficiency [11]. Additionally, optimizing light utilization by reducing spot size contributes to improving the SNR. However, reducing laser spot size requires more sampling point data, which may reduce detection efficiency. From a system design perspective, the incident angle of the laser and the viewing angle of the detectors are crucial factors for achieving optimal scattering distribution, which is critical for detection efficiency [12]. In addition, exploiting phase [13] and polarization information [14] of the light has proved effective in detecting smaller features, thereby improving defect detectability. A high-numerical-aperture (NA) objective lens can collect enough light and improve the SNR, but limit field of view (FOV) and detection efficiency [15]. From a photodetector perspective, on the one hand, point scanning represented by a photomultiplier tube (PMT) has high scanning efficiency. Currently, non-patterned wafer detection mainly relies on rapid point scanning via PMT [16]. However, the multi-stage amplification of the PMT makes it sensitive to light so that it tends to falsely judge the haze signal as a defect signal [17,18]. In addition, this non-imaging scan requires data conversion from one dimension to two dimensions (2D) according to the scanning path. On the other hand, areal scanning via a Charge-Coupled Device (CCD) or a Complementary Metal Oxide Semiconductor (CMOS) produces 2D images that are more intuitive and have lower noise levels. However, sub-aperture stitching is relatively complex and time consuming [19,20,21,22]. It is not suitable for detecting defects in motion environments because of motion blur. Table 1 provides a comparison of the point scanning mode and the areal scanning mode. In addition, from an algorithmic perspective, image filtering, image enhancement, and morphological algorithms are frequently employed to enhance and extract defect signals, consequently improving the SNR [23]. Machine learning [24] and deep learning [25] are mainly used for defect recognition and classification to improve detection sensitivity and specificity. However, algorithm complexity also affects detection efficiency.

It is worth noting that TDI image sensors offer several features such as noise suppression, a larger FOV, and fast imaging speed, which combine the advantages of PMT and CCD. The TDI feature allows a longer exposure time while maintaining a high frame rate, resulting in higher sensitivity of the image sensor and images with high contrast and low noise. These characteristics make TDI image sensors suitable for dark-field microscopy. Currently, TDI research is used for patterned wafer inspection via bright-field microscopy [26] and biological sample observation via fluorescence microscopy [27,28,29]. However, bright-field and fluorescence microscopy have limitations for non-patterned wafer inspection due to high reflection and contamination.

This paper provides a method to improve the SNR of defects without sacrificing efficiency, thus achieving a balance between detection efficiency and sensitivity. The potential benefits, limitations, and practical considerations are discussed through a comprehensive analysis of experimental results and comparative studies. It provides targeted guidance and suggestions for wafer inspection in production lines. The paper is structured as follows. Section 2 describes the principle of dark-field scattering and TDI imaging. Section 3 describes the detailed information on TDI-based dark-field scattering microscopy (TDI-DFSM). Section 4 presents the experimental results and discussions. Finally, Section 5 is the summary of this paper.

## 2. Methodology

### 2.1. Dark-Field Scattering

**Figure 2:**
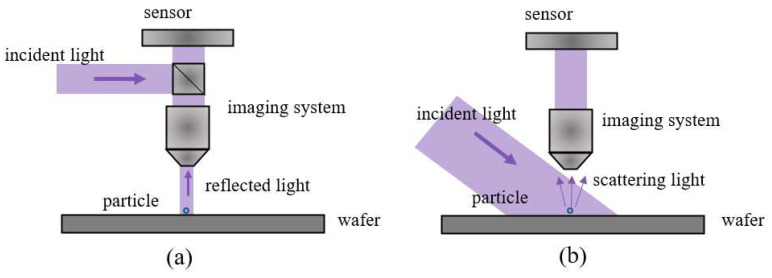
Schematic of wafer defect scattering: (**a**) bright field; (**b**) dark field.

**Figure 3:**
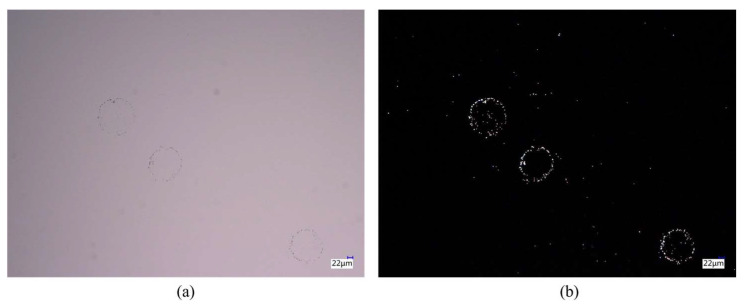
Particles on the non-patterned wafer via KEYENCE VHX-5000: (**a**) captured by bright field; (**b**) captured by dark field.

Dark-field scattering has emerged as a powerful microscopy technique for detecting and characterizing microscopic particles, structures, and defects. Unlike bright-field microscopy, which illuminates the sample under test with direct light resulting in a bright background, dark-field microscopy employs oblique illumination [30]. Figure 2 illustrates the principle of dark-field and bright-field scattering imaging. The wafer surface is illuminated obliquely by incident light. Complete reflection of the light occurs when there are no defects on the wafer surface. In the presence of defects, the microscopy system collects scattering light, resulting in an image of bright defects against a dark background [31]. This imaging technique can improve the visualization and characterization of small, low-contrast defects that may be difficult to discern using bright-field microscopy [32]. Figure 3 shows the comparison of particles on the non-patterned wafer under bright-field and dark-field conditions via KEYENCE VHX-5000 (Keyence Corporation, Osaka, Japan). Particle scattering on wafer surfaces is complex and its rigorous solution is difficult to obtain. However, it can be expressed qualitatively as [33]

(1) $$I_{p}\propto I_{o}\frac{d^{6}}{\lambda^{4}}$$

where $I_{p}$ represents the particle scattering light intensity, $I_{o}$ represents the incident intensity, *d* represents the particle size, and *λ* represents the incident wavelength. Short-wavelength and high-power light sources can effectively increase the intensity of the scattering signal intensity from particles, thereby increasing defect detectability. Meanwhile, the lens transmittance problem at a short wavelength and laser-induced wafer damage at high power also need to be considered.

### 2.2. Fundamental Aspects of TDI

**Figure 4:**
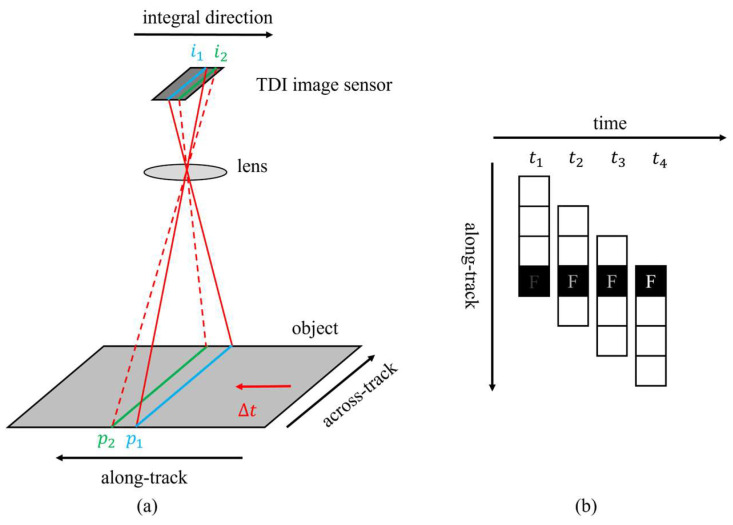
Fundamental aspects of TDI: (**a**) the imaging process of the TDI imaging system; (**b**) the character of TDI (TDI stage is 4 as an example).

A TDI image sensor is a kind of special line-scanning image sensor that captures image information through pixels arranged in an array and working in a line-scanning mode. The imaging principle of the TDI image sensor is illustrated in Figure 4. In Figure 4a, the object moves to the left and the integral direction of the TDI image sensor is to the right. At a certain moment, object position p_1_ is imaged at image position i_1_. When the object moves after ∆t time, position p_1_ moves to p_2_, and the corresponding image position i_1_ moves to i_2_. Meanwhile, the charge at position i_1_ is transferred to position i_2_ and accumulated. Finally, the entire image in the along-track direction is acquired with the movement of the object. Figure 4b shows that the contrast of the image captured at the same position improves over time. During multiple delay integrations, it is beneficial to obtain higher SNR images in low-light intensity environments.

In general, TDI image sensors consist of *N* TDI stages. As the object under test moves, the TDI image sensor sequentially captures light from the first stage to the *N*th stage, and the charge accumulates from the first stage to the *N*th stage. The accumulated charge is then transferred to the readout channel after multiple delay integrations. Finally, the digital signal is output through the programmable gain amplifier and the analog-to-digital converter. For the TDI image sensor, the exposure time and the SNR can be expressed as follows, respectively:

(2) $$t=\frac{N}{F}$$

(3) $$SNR=\frac{S}{\sigma }=\frac{nt\eta g}{\sigma }=\frac{nN\eta g}{F\sigma }$$

where *S* represents the signal intensity, *σ* represents the background noise, *n* represents the number of photons received per pixel per unit time, *t* represents the exposure time, *η* represents the quantum efficiency, *g* represents the sensor gain, and *F* represents the line frequency of the TDI image sensor. The relationship between *SNR* and line frequency *F* reveals an approximately negative correlation. To optimize detection efficiency without sacrificing the SNR, it is necessary to increase the values of *n* and *N* and decrease the value of *σ*. Although the significance of TDI sensor noise is acknowledged, it is noteworthy that increasing the number of TDI stages yields a more substantial signal gain compared to the accompanying background noise. Ideally, increasing the number of TDI stages by a factor of *m* is expected to improve the SNR by *√m*. This is an effective strategy for enhancing overall system performance.

## 3. Experimental Setup

**Figure 5:**
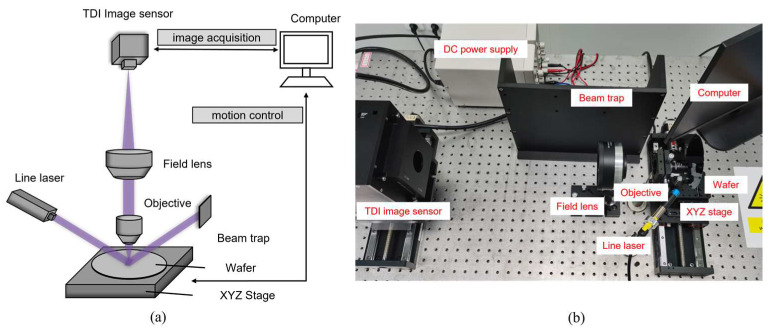
TDI-based dark-field scattering microscopy: (**a**) schematic diagram; (**b**) experimental device.

To analyze and verify the efficiency and sensitivity of TDI in dark-field inspection, the TDI-DFSM is built as a fundamental platform. Its schematic diagram and experimental device are shown in Figure 5. The detailed specifications of the TDI image sensor used (Gpixel GLT5009BSI) are shown in Table 2. The number of pixels along the across-track direction is 9072, and the number of TDI stages along the along-track direction is 256. A line laser (MZlaser MZM12405100L30) with a wavelength of 405 nm and a power of 100 mW is selected to irradiate the wafer surface at an angle of 60 degrees. Collecting as much scattering light as possible to the exclusion of reflected light is a major factor in the selection of the angle of incidence. The laser beam is modulated into a uniform flat-topped beam to ensure the same energy density across the illuminated area. To reduce the effect of stray light, a beam trap absorbs the reflected light. An objective lens (Thorlabs LMU-15X-NUV, NA 0.3) receives the scattering light from the defect, which then enters the TDI image sensor via a tube lens. However, the physical dimensions of the sensor limit its utilization in the microscopy system. Traditional tube lenses (*f* = 150 mm or 200 mm) do not fully utilize the large FOV of the TDI image sensor. To address this constraint and maximize the performance of the TDI image sensor, a field lens with *f* = 330 mm (Carman Haas SL-355-220-330) is considered to replace the tube lens. Therefore, the magnification of the entire microscopy imaging system is $f_{FL}/f_{OL}=330/14.1\approx 23.4$. In addition, a computer is utilized to control line frequency, sensor gain, TDI stages, and other adjustable register parameters of the TDI image sensor for image acquisition. Furthermore, the computer controls the XYZ stage to achieve wafer motion. The maximum motion speed of the object is 10 mm/s.

Imaging quality is affected by multiple factors such as illumination conditions, mechanical movement, and imaging systems. To ensure that the 2D image captured by the TDI image sensor is not blurry due to the rapid motion of the object, the line frequency needs to match the wafer movement speed, and the direction of charge accumulation needs to be opposite to the motion direction of the wafer. Thus, the image has the same resolution in the x-direction and the y-direction. We set the line frequency of the TDI image sensor according to the following equation:

(4) $$\frac{L_{o}}{H_{c}}=\frac{V_{o}}{F}$$

where $L_{o}$ is the FOV width, $H_{c}$ is the number of pixels in the across-track direction, and $V_{o}$ is the motion speed of the object. The diffraction-limited resolution is calculated as 0.61λ/NA=0.61×0.405/0.3≈0.823 μm according to the Rayleigh criterion and the Nyquist sampling law. In addition, the system achieves a detection sensitivity of defect size 0.5 μm, and possibly even smaller ones. This is described in Experimental Section.

## 4. Experimental Results and Discussions

### 4.1. Linearity Assessment of the TDI Image Sensor

**Figure 6:**
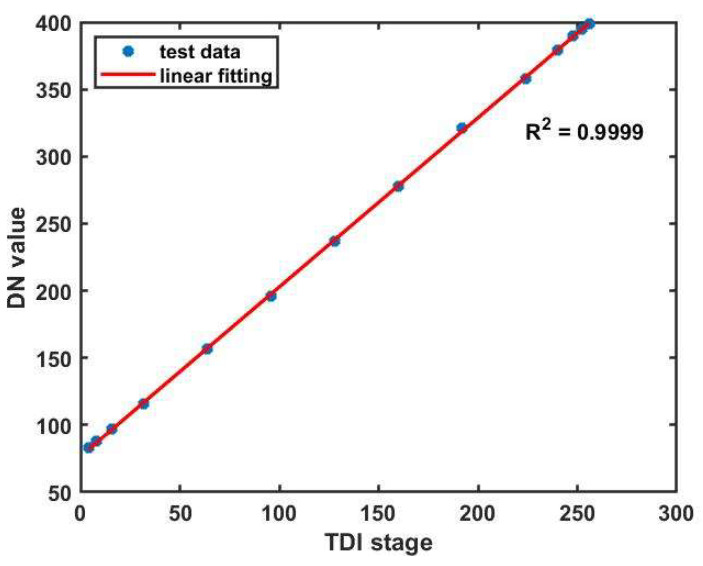
Linearity of the TDI image sensor.

The linearity of the TDI image sensor affects the imaging results. Figure 6 shows the relationship between the output signal (Digital Number, DN value) and the TDI stages under fixed illumination conditions. The line frequency and the sensor gain are constant. The test data are obtained by shining a circular white source onto a non-patterned wafer without defects. The linear fit metric is calculated according to the following equation:

(5) $$R^{2}=1-\frac{\sum_{i}{\left(y_{i}-\hat{y}\right)}^{2}}{\sum_{i}{\left(y_{i}-\bar{y}\right)}^{2}}$$

where $y_{i}$ represents the DN value at different TDI stages, $\bar{y}$ represents the mean DN value, and $\hat{y}$ represents the estimated value by the least square method. The result illustrates the excellent linearity and confirms its capability to ensure consistent pixel response across different rows for the same position of a moving object.

### 4.2. Resolution Target Imaging

**Figure 7:**
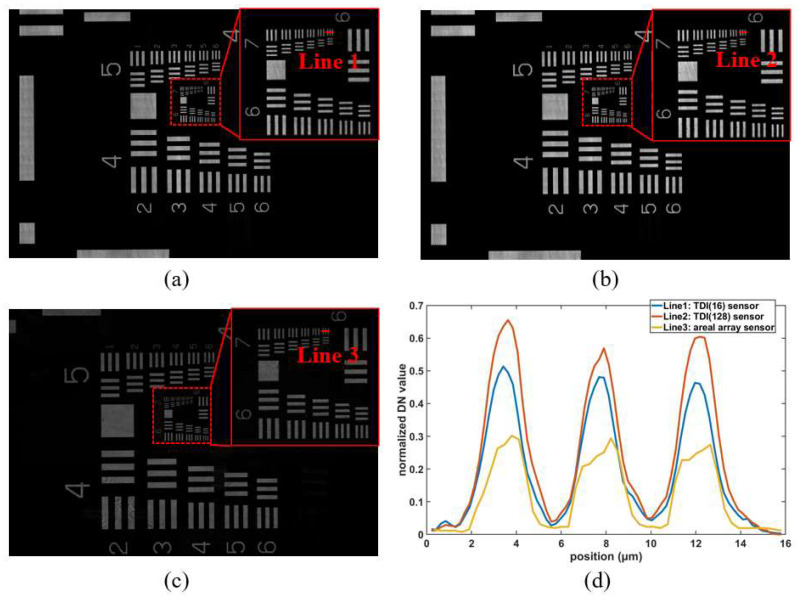
USAF 1951 target images captured by the TDI image sensor and the areal array sensor, respectively: (**a**) TDI sensor (TDI stage 16); (**b**) TDI sensor (TDI stage 128); (**c**) areal array sensor; (**d**) the normalized DN values of three lines.

Figure 7 shows the USAF 1951 target images captured by the TDI image sensor and images captured by the areal array sensor (Pike F-1600C model) for comparison. All sensors work in non-saturation and have the same gain. The USAF 1951 target is illuminated vertically with fixed low-power incident light to avoid overexposure. Figure 7a,b are captured by the TDI image sensor at 16 TDI stages and 128 TDI stages, respectively. The exposure time is 1.4 milliseconds and 11 milliseconds, respectively, according to Equation (2). The horizontal motion speed of the USAF 1951 target is 2.5 mm/s to capture all details, and the line frequency is set to 11,700 Hz according to Equation (4). Figure 7c is captured by the areal array sensor with the same exposure time of 11 milliseconds as in Figure 7b. Under conditions of object motion, images captured by the areal array sensor can produce severe motion blur, while the TDI sensor has a significant advantage in capturing moving objects. Figure 7d shows the normalized intensity values along a specified line segment direction (perpendicular to the direction of the stripes). The number of acquisition points of the three traces is 74, 74, and 50, respectively. The TDI sensor demonstrates superior signal intensity compared to the areal array sensor, even with the utilization of only 16 TDI stages. Furthermore, it is evident that increasing the number of TDI stages significantly enhances the overall imaging performance.

### 4.3. Particle Inspection on Non-Patterned Wafer

**Figure 8:**
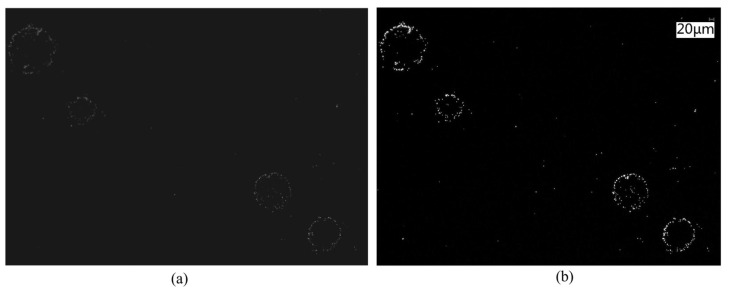
Comparison of particle inspection: (**a**) captured by the TDI-DFSM; (**b**) captured by KEYENCE VHX-5000.

To inspect defects on the non-patterned wafer, 0.5 μm polystyrene latex (PSL) is employed to mimic particle defects. These PSLs are diluted and sprayed onto the polished non-patterned wafer. Due to the self-aggregation phenomenon of PSLs, the captured images show multiple microspheres gathering in a limited area, as shown in Figure 8a. The horizontal motion speed of the wafer is 5 mm/s to test the defect detection capability at higher speed, the line frequency of the TDI image sensor is set to 23,400 Hz, and the TDI stage is set to 32. To determine the detection capability of the TDI-DFSM, the comparison results with KEYENCE VHX-5000 are shown in Figure 8b. Excluding considerations for the four aggregation regions and focusing on the count of individual PSL particles, both systems record counts of 60. This demonstrates that the TDI-DFSM has a 100% detection capability for 0.5 μm spherical particles, with no false detections or missed detections.

**Figure 9:**
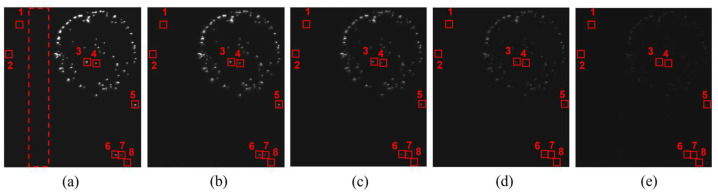
Particles in the same wafer area captured at different TDI stages: (**a**) TDI stage 256; (**b**) TDI stage 192; (**c**) TDI stage 128; (**d**) TDI stage 64; (**e**) TDI stage 16.

**Figure 10:**
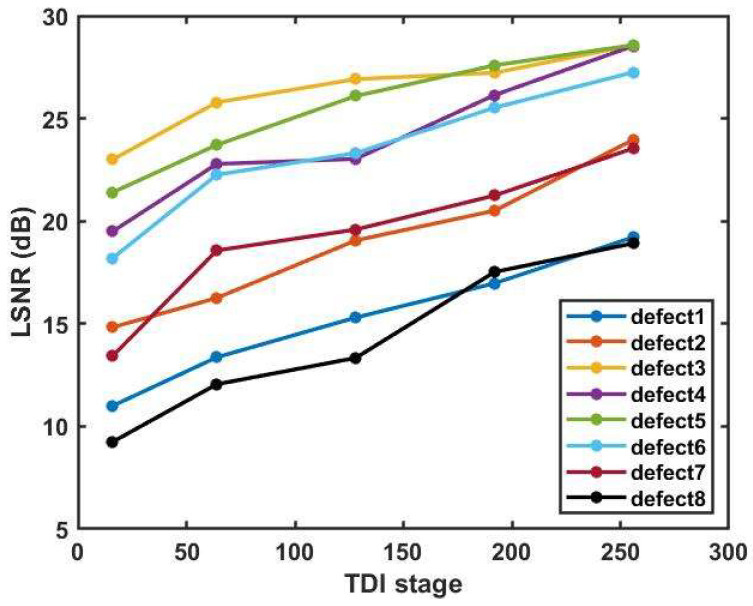
LSNR of the particles numbered 1–8 in Figure 9 at different TDI stages.

To investigate the impact of TDI stages on dark-field scattering defect inspection, the same wafer area is imaged under fixed illumination conditions and different TDI stages, as shown in Figure 9. The object motion speed and sensor gain are constant. To ensure reasonable data distribution and effective data differentiation, several typical TDI stages for observing particle defects are chosen. The local signal-to-noise ratio (LSNR) is defined as a quantitative measure of the signal quality relative to the background noise. Specifically, it is defined as follows:

(6) $$LSNR=10\times \log_{10}\frac{\left|S_{defect}-S_{back}\right|}{\sigma_{back}}$$

where $S_{defect}$ represents the mean value in a 3 × 3 neighborhood centered on the maximum value of the defect signal at different TDI stages, $S_{back}$ and $\sigma_{back}$ represent the mean value and the standard deviation of the background image without defects in the red dashed area at different TDI stages, respectively. Table 3 shows the experimental results of background means and background standard deviations. Figure 10 shows the LSNR of the particles numbered 1–8 in Figure 9 at different TDI stages. The LSNR of the particles improves significantly with increasing TDI stages. Similar results are obtained through multiple experiments. Without considering the data transmission time, the imaging time is only related to line frequency. Therefore, the increase in the number of TDI stages does not affect the line frequency, thus improving the SNR without sacrificing detection efficiency.

**Figure 11:**
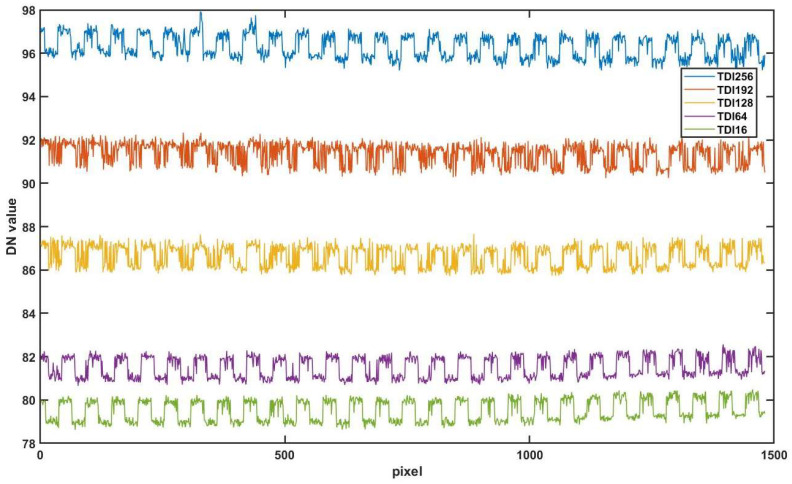
Average CFPN distribution of background images at different TDI stages.

It should be noted that in Figure 9e, when the signal intensity of particles is weak at low TDI stages, the presence of stripe-like column fixed pattern noise (CFPN) in the background can impact particle detection. This CFPN is caused by a mismatch between the readout circuits of different columns. Although the correlated dual sampling circuit structure is an effective method to eliminate CFPN, this elimination is incomplete. Figure 11 shows the average CFPN distribution of background images. It is obtained by calculating the average DN value of 200 rows of pixels in the defect-free red dashed area corresponding to different TDI stages in Figure 9. Changing the number of TDI stages does not significantly alter the distribution of CFPN, but only varies in the amplitude of the background signal. Image difference is a simple and effective method to reduce the CFPN. The background images without particles are subtracted from the particle images taken at different TDI stages. Table 4 shows the standard deviations of background images at different TDI stages after CFPN reduction, which are reduced by almost 50% compared to the raw standard deviation without processing in Table 3. The noise reduction contributes to SNR improvement, especially for images taken at low TDI stages.

In the experiment, the line frequency is fixed at 23,400 Hz, allowing calculation of the imaging efficiency as $V_{o}L_{o}=5\times 1.93\approx 10{\;\mathrm{mm}}^{2}/s$. The scanning time for an 8-inch wafer is determined as ${\left(2r\right)}^{2}/V_{o}L_{o}={\left(2\times 100\right)}^{2}/10\approx 1.12\;h$. For practical applications in production lines, utilizing a deep ultraviolet (DUV) source with a wavelength of 193 nm, the resolution is calculated as 0.61λ/NA=0.61×0.193/0.3≈0.392 μm. Line illumination different from point focusing can avoid laser-induced wafer damage and provide space for the growth of laser power. High-power, short-wavelength lasers can shorten exposure time, thereby improving the line frequency of the TDI image sensor and detection efficiency. Additionally, optimizing line frequency to its maximum value of 608 kHz results in a scanning speed of 129 mm/s, reducing the scanning time to just 3 min for an 8-inch wafer. In summary, Table 5 summarizes the key specifications of the TDI-DFSM and its potential application parameters in production lines.

It should be noted that the system is an experimental verification device, and it can be further enhanced for practical applications. The uniformity of the illumination light and the intensity of the scattering light can be increased by setting up ring light sources. The combination of a circular scanning strategy and multiple dark-field scattering acquisition channels offers the possibility of improving detection efficiency. However, the different line frequencies of the TDI image sensor need to be adjusted to accommodate linear velocity changes at different radii, thereby eliminating image blur. The TDI-DFSM can integrate an autofocus mechanism, an auto-calibration procedure, and a feedback mechanism, thus reducing manual intervention and improving system stability. Therefore, the relationship between defocus distance and signal intensity needs to be established. In addition, the TDI image sensor can capture multiple images via a single scan, improving data throughput and defect detectability. Polarization modulation and ellipsometry techniques can provide more information and are expected to improve detection sensitivity. In microscopy systems, the depth of field is limited, and wafer movement during inspection inevitably leads to defocusing issues. However, image quality can be improved using blind deconvolution algorithms or generative adversarial networks. Due to defocusing causing a decrease in sensitivity, the depth of field may limit the detection of the texture or bulk defects that are buried in penetrable environments such as SiC.

## 5. Conclusions

In summary, a TDI-DFSM with a FOV of 1.93 mm, a detection efficiency of 10 mm^2^/s, and a sub-micron detection sensitivity was developed. A field lens replaced the traditional tube lens to fully cover a large photosensitive area. The TDI-DFSM achieved multi-point simultaneous scanning, which is different from the PMT. In addition, by increasing the number of TDI stages and reducing the CFPN, the SNR of defect images was effectively improved without sacrificing detection efficiency, providing a reasonable balance between detection efficiency and sensitivity. With the support of appropriate light sources, motion mechanisms, and computer computing power, the potential configuration and detection efficiency of TDI-DFSM will achieve remarkable improvement. It has promising applications for non-patterned wafer inspection in production lines. Future work will focus on utilizing polarization information, optimizing scanning strategy, and applying deblurring algorithms, which will further improve the detection sensitivity and efficiency of the TDI-DFSM.

## Figures and Tables

**Table 1:** Comparison of the point scanning mode and the areal scanning mode.

Scanning Mode	Advantages	Disadvantages
point scanning	high speed	noise-sensitive, 1D signal
areal scanning	low noise	low speed, image stitching

**Table 2:** Specifications of the TDI image sensor.

Parameter	Parameter Value
photosensitive area	45.36 mm × 1.28 mm
pixel size	5 μm × 5 μm
number of active pixels	9072 × 256
max line frequency	608 kHz
ADC depth	10 bits
available TDI stages	4, 8, 16, 32, 64, 128, 160, 192, 224, 240, 248, 252, 256

**Table 3:** Quantitative measures at different TDI stages.

TDI Stage	16	64	128	192	256
means (DN)	79.5188	81.5049	86.6343	91.3814	96.2994
standard deviation (DN)	0.8236	0.8825	1.0533	1.1870	1.2856

**Table 4:** Standard deviations of background images at different TDI stages after reducing CFPN.

TDI Stage	16	64	128	192	256
standard deviation (DN)	0.3937	0.4419	0.5527	0.6279	0.6853

**Table 5:** Key specifications of the TDI-DFSM and its potential application parameters in production lines.

Specification	TDI-DFSM Parameter	Application Parameter
laser wavelength	405 nm	248 nm or 193 nm
laser power	100 mW	several/tens of watts
resolution	0.823 μm	0.392 μm
detection sensitivity	0.5 μm	<0.392 μm
line frequency	23,400 Hz	608 kHz
scanning speed	5 mm/s	129 mm/s
imaging efficiency	10 mm^2^/s	249 mm^2^/s
scanning time for 8-inch wafer	1.12 h	3 min

## Data Availability

Data are contained within the article.
